# Systemic Therapy of Non-Resectable Metastatic Melanoma

**DOI:** 10.3390/cancers2020955

**Published:** 2010-05-26

**Authors:** Azadeh Orouji, Sergij Goerdt, Jochen Utikal

**Affiliations:** Department of Dermatology, Venereology and Allergology, University Medical Center Mannheim, Ruprecht-Karl University of Heidelberg, Mannheim, Germany; E-Mails: azadeh.orouji@umm.de (A.O.); sergij.goerdt@umm.de (S.G.)

**Keywords:** melanoma, metastatic, chemotherapy, targeted therapy, small molecules

## Abstract

In advanced metastatic melanoma (non-resectable stage III/IV), the prognosis still remains poor, with median survival times between six and twelve months. Systemic therapeutic approaches for metastatic melanoma include chemotherapy, immunotherapy, immunochemotherapy, small molecules and targeted therapy. In this review, we will focus on the various treatment modalities as well as new agents used for targeted therapy.

## 1. Introduction

Melanoma is an aggressive tumor with a continuously growing incidence worldwide. Depending on tumor thickness, ulceration and lymphocytic infiltration at the time of diagnosis, 20–25% of all primary melanoma will spread [1,2]. In case of distant metastatic melanoma, with the exception of rare cases of surgery for oligometastatic disease, the prognosis is fatal with median survival times between six and twelve months. It has a three-year survival rate of only 10–15% [3]. Here we summarize therapeutic options in distant metastatic disease.

## 2. Chemotherapy

In cases of non-resectable metastatic melanoma, systemic chemotherapy is a therapeutic option, besides radiotherapy. Chemotherapeutic agents are cytotoxic anticancer drugs that impair cell division, resulting in the death of rapidly dividing cells.

Single-agent chemotherapy produces responses in 10–25% of patients with advanced melanoma, although there is no evidence that this translates into a survival advantage. We see complete responses only in about 2% of the cases. The median survival associated with chemotherapy is nine months. 13% of patients are alive at two years [4]. Compounds including dacarbazine (DTIC), temozolomide, fotemustine and vindesine are commonly used (see Table 1). 

**Table 1:** Cytotoxic drugs used in the therapy of distant metastatic melanoma.

Chemotherapy	
**Single-agent chemotherapy**	
Dacarbazine (DTIC)	200–250 mg/m^2^/day i. v., 5 daily doses 800–1200 mg/m^2^ i. v., every 3–4 weeks
Temozolomide	150–200 mg/m^2^ p. o. 5 daily doses (day 1–5), every 4 weeks 150 mg/m^2^ p. o., day 1–7, then 7 days pause (biweekly)
Fotemustine	100 mg/m^2^ i. V. day 1 and 8 and 15, then 5 weeks pause, continuation every 3 weeks
Vindesine	3 mg/m^2^ i. v., every 2 weeks
**Combination chemotherapy**	
Carboplatin/ Paclitaxel	Carboplatin AUC 6 i. v. day 1 Paclitaxel 225 mg/m^2^ i. v. day 1 every 3 weeks
Gemcitabine/ Treosulfan	Gemcitabine 1000 mg/m^2^ i. v. day 1 and 8 Treosulfan 3500 mg/m^2^ i. v. day 1 and 8 every 3 weeks
BHD	BCNU 150 mg/m^2^ i. v. day 1, every 2 cycles Hydroxyurea 1500 mg/m^2^ p. o. day 1–5 Dacarbazine 150 mg/m^2^ i. v. day 1–5 every 4 weeks
BOLD	Bleomycin 15 mg/m^2^ i. v., day 1 and 4 Vincristine 1 mg/m^2^ i. v. day 1 and 5 CCNU 80 mg/m^2^ p. o. day 1 Dacarbazine 200 mg/m^2^ i. v. day 1–5 every 4 weeks
DVP	Dacarbazine 450 mg/m^2^ i. v., day 1 and 8 Vindesine 3 mg/m^2^ i. v., day 1 and 8 Cisplatin 50 mg/m^2^ i. v., day 1 and 8, every 3–4 weeks

### 2.1. Dacarbazine (DTIC)

In 1975, dacarbazine (DTIC) became the first US Food and Drug Administration (FDA) approved chemotherapeutic agent for the treatment of metastatic melanoma. As a single agent, dacarbazine was most commonly used even when it had not been formally compared with other agents or with observation alone. The usual dose is 800–1200 mg/m^2^ every 3–4 weeks (given either in one dose or in five doses of 200–250 mg/m^2^ on different days). The response rates with dacarbazine are 15–25%, with the median response duration of 5–6 months, but less than 5% of complete responses. Long-term follow-up of patients treated with dacarbazine alone shows less than 2% survival rate for six years [5,6].

### 2.2. Temozolomide

Dacarbazine can also be substituted with temozolomide, an orally available analog of dacarbazine, because of its convenience of adminstration. This substance has the advantage of central nervous system penetration. Temozolomide has shown an equal efficacy to that of dacarbazine in a phase III trial at a dose of 200 mg/m^2^/day for five days every 28 days. Recent results from a large, randomized phase III trial showed no differences in overall survival, progression-free survival and overall response rate between temozolomide arm and dacarbazine arm. This trial examined the efficacy of an extended schedule of temozolomide (week on–week off, 150 mg/m^2^/day for seven days, repeated every 14 days) compared with standard dose single-agent dacarbazine. Temozolomide was very well tolerated and showed an improvement in the quality of life, resulting in large use of temozolomide for the treatment of metastatic melanoma [7]. 

### 2.3. Fotemustine

Fotemustine, a nitrosourea, is probably the most active one of this group in metastatic melanoma. The nitrosoureas can cross the blood-brain barrier. Fotemustine has been widely tested in Europe and has shown an overall response of 20–25%, including 5–8% of complete response rates. It was the first drug showing significant efficacy in brain metastases [8,9]. In a study comparing foremustine to dacarbazine, fotemustine produced a higher overall response rate than dacarbazine. However, response duration (time to disease progression and overall survival) was not statistically significant. Interestingly, in patients without brain metastasis at inclusion, the median time to brain metastasis was longer in fotemustine comparing to dacarbazine (22.7 months *versus* 7.2 months). This trial showed no significant difference in terms of quality of life between both arms [10].

### 2.4. Vindesine

Vindesine is a vinca alkaloid used as a single agent therapy as well as in polychemotherapy in patients with metastatic melanoma. The vinca alkaloids block cell division in metaphase. The main side effects of vindesine are nephrotoxicity and neurotoxicity. A mild myelosuppression is another side effect of vindesine [11].

### 2.5. Polychemotherapy

The poor efficacy of the single agent chemotherapy led to the evaluation of polychemotherapy in the 1980s to improve outcome and enhance response rates in patients with metastatic melanoma. The first combinations added nitrosourea, vinca alkaloids or platinum to dacarbazine, failing to result in any significant benefit compared with dacarbazine alone, except for a slight increase of response rates. Other more aggressive polychemotherapy regimens such as BHD (BCNU, hydroxyurea, and dacarbazine) and BOLD (bleomycin, vincristine, CCNU, and dacarbazine) were used, and resulted in higher response rates without any survival advantage. In a phase II trial with the combination of dacarbazine, cisplatinum and vindesine (DVP or CVD), a response rate of 40% was obtained, including 4% of complete response [12]. In a randomized study on 150 patients comparing CVD to dacarbazine, the response rate was higher in the CVD arm as compared to the dacarbazine arm (19% to 14%), without any differences in either response duration or survival. However, increased toxicity has been observed [13]. The combination of tamoxifen and dacarbazine was tested in a small phase III study. It demonstrated an improvement of response and survival compared to dacarbazine alone [14]. However, other large randomized studies did not support this result. The reported efficacy of the combination of paclitaxel and carboplatin used in the therapy of metastatic melanoma in recent years was one of the more unexpected developments.

The other chemotherapy combination is gemcitabine and treosulfan. To evaluate an *in vitro* test system providing information on the drug sensitivity profile of melanoma cells, Ugurel *et al.* examined tumor tissue specimens from metastatic melanoma patients with an ATP-based chemosensitivity assay (ATP-TCA). In the chemosensitivity testing in 31 metastatic melanoma patients, the highest sensitivity was detected for this combination. 76% of the tissue samples revealed high sensitivity and 10% resistance [15]. Chemosensitive patients showed an increased overall survival of 14.6 months compared with 7.4 months in chemoresistant patients [16].

Huncharek *et al*. reported in a meta-analysis comparing two or three drug combination regimens with dacarbazine alone, that there was no advantage for the combination chemotherapy in terms of response or survival. Treatment decisions remain controversial, and quality of life and toxicity issues from treatment assume greater importance [17]. 

As a first-line therapy, polychemotherapy did not show significant advantages for prolongation of survival compared to single agent therapy; hence it is more toxic. As treatment in metastatic non-operable malignant melanoma is primarily palliative, the effect of any regimen on the quality of life must be carefully weighed [18].

## 3. Immunotherapy

The Term “immunotherapy” is used for non-specific as well as specific immunomodulation that encompasses a number of different approaches, summarized below.

### 3.1. Cytokines

The most widely used immunomodulating drugs in metastatic melanoma are Interleukin 2 (IL-2) and Interferon α (IFN-α). An overall objective response rate of 16% and a complete response in 6% of patients treated with high-dose IL-2 have been reported [19]. IL-2 can cause severe hypotension and vascular leak syndrome, resulting in interstitial and pulmonary edema, renal and hepatic dysfunction, cardiovascular failure, neurological disturbances, nausea, vomiting and thrombocytopenia [20]. The significant toxicity of the treatment with IL-2 (Proleukin) has limited its use to selected patients with good organ function who are treated by experienced clinicians at selected specialized centers [2]. 

Numerous studies have demonstrated that IFN-α has antiproliferative and immunomodulatory effects, including the inhibition of angiogenesis [21], the increase of major histocompatibility complex class I antigen expression and the infiltration of CD^4^+ T cells into melanomas [22]. In a metastatic situation, single agent IFN-α showed approximately 15% of responses with less than 5% of complete response rates and median response duration between six and nine months, with a maximum of twelve months for the best studies [23]. These response rates, while encouraging, were not significant enough to lead to its widespread use in the treatment of metastatic melanoma. However, observations that patients with non-visceral disease were more likely to response suggested that the use of IFN-α may demonstrate a greater impact in patients with micrometastasis [23,24]. 

### 3.2. Monoclonal Antibodies

Anti-cytotoxic T Lymphocyte-Associated Antigen 4 (CTLA-4)

Activation of naïve T cells requires recognition of the antigen by the T cell receptor (TCR) and provision of costimulatory signals. CTLA-4 is expressed on the surface of Helper T cells and transmits an inhibitory signal to T cells. The pivotal role of CTLA-4 in regulating T-cell function is established, and a series of preclinical studies provided proof-of-concept evidence of the antitumor activity of anti-CTLA-4 antibodies in combination with vaccines or chemotherapy. Subsequently, anti-CTLA-4 antibodies have shown encouraging results in clinical trials in advanced melanoma. Recent progress in the understanding of melanoma genetics and tumorgenesis has led to potential new therapeutic targets. Molecular targeted agents that inhibit the proliferation and survival of metastatic melanoma cells offer potential partners for anti-CTLA-4 antibodies in combined modality regimens [25]. Two monoclonal antibodies recognizing CTLA-4, ipilimumab (MDX 010) and tremelimumab (CP-675, 206), have been examined in many trials. 

Activity of ipilimumab in patients with metastatic melanoma has been examined with and without chemotherapy. In a randomized phase II trial, ipilimumab was given alone or in combination with dacarbazine. The median overal survival (OS) for the monotherapy group was 351 and for the combination group 389 days, respectively. Approximately 10% of the patients were alive after two to more than four years of follow-up in both therapy arms. This fact indicates that the therapy can achieve a long-term control of the disease. 

The activity of ipilimumab in combination with a vaccine (two modified HLA-A* 0201.restricted peptides) was tested in a randomized study (phase II) in 56 patients [26]. The overall response rate was 13%. Patients with grade 3/4 autoimmune toxicity show better clinical responses. It is reported that some of the immune-related adverse events were observed in 62% of 139 overall treated patients and were associated with a greater probability of objective antitumor response [27]. Colitis or diarrhea and also dermatitis were the most common grade 3/4 immune-related adverse events [28,29,30]. The induction of manageable autoimmunity in patients with metastatic melanoma treated with ipilimumab could be a marker of objective and durable clinical response.

Tremelimumab (CP-675, 206) is another fully human monoclonal antibody specific for human cytotoxic T lymphocyte-associated antigen 4 (CTLA-4, CD 152) in clinical development for patients with cancer [31]. In a single-arm phase II trial with 246 previously treated patients, assessing the activity of tremelimumab as a single agent shows the objective response rate of only 6.6%. The duration of response was 8.9 to 29.8 months. Clinical benefit rate (overall response + stable disease) was 21% (16 partial responses and 35 stable disease), and median overall survival was 10.0 months. Progression-free survival at six months was 15%, and survival was 40.3% at 12 months and 22% at 24 months. This indicates a role for tremelimumab in these patients [32]. A randomized study investigating tremelimumab as a single agent in comparison with a standard chemotherapy (decarbazine or temozolomide) in previously untreated patients concluded that the antibody failed to demonstrate an improvement in OS as a first-line treatment in patients with metastatic melanoma [33]. An overall response rate of 19% for a combination therapy with tremelimumab and high-dose Interferon-α2b was shown in a phase II trial of 16 previously treated patients [34]. Early-Phase clinical trials demonstrated acceptable toxicity of tremelimumab (diarrhea/colitis, dermatitis, fatigue, pancreatitis, Grave’s disease) [35].

### 3.3. Vaccines

In trials involving 440 metastatic cancer patients (96% melanoma) receiving cancer vaccine, the objective response was very low (2.6%). The other 40 studies with 756 patients have showed a comparable result of 4.0% response rate [36]. It is discussable that the vaccines may only be suitable for immune-competent patients (after full resection of their tumors, adjuvant setting). However, large adjuvant trials after tumor resection (stage II-IV melanoma) have not demonstrated positive results. 

#### 3.3.1. Allogeneic and Autologous Vaccines

The autologous vaccines are prepared from tumors of individual patients. Vitespen is a tumor-derived, HSP-peptide complex vaccine that was tested in a study with 64 metastatic melanoma patients. A phase III study with 322 previously untreated metastatic melanoma patients compared Vitespen with a physician choice therapy. A similar overall survival (OS) was found in both arms [37]. In two large trials, more than 1400 patients were randomized to Canvaxin + BCG (bacillus Calmette-Guerin) or placebo + BCG after resectional surgery. The five-year survival rate in stage III was 59% for the Canvaxin patients and 68% for the placebo patients. In the stage IV study, the median survival was 32 months for the Canvaxin patients and 39 months for the placebo patients. The five-year survivals were 40% (Canvaxin) and 45% (placebo) [38,39]. 

#### 3.3.2. MAGE-3

Tumor regression in melanoma patients has been documented in several trials involving antigens encoded by genes of the MAGE family, particularly MAGE-3. Low-level cytolytic lymphocyte (CTL) responses mediate these tumor regressions [40]. A randomized, open trial with the MAGE-A3 protein combined with different immunological adjuvants- AS02B or AS15- assessed the adjuvants for toxicity and clinical and immunological responses. 68 patients with unresectable stage III or stage IV M1a melanoma got the MAGE-A3 protein as first-line treatment. In combination with AS15, it yielded higher anti-MAGE-3 antibody titer, stronger T cell induction and long-lasting clinical response [41]. 

## 4. Small Molecules and Targeted Therapies

Another interesting starting point in the therapy of metastatic melanoma is the selected blocking of targeted structures. Several signal pathways related to survival of cancer cells are frequently deregulated in melanoma (RAS-RAF-MEK-ERK pathway, p16INK4A-CDK4-RB pathway, ARF-p53 pathway, PI3K-AKT pathway and the canonical Wnt signaling pathway). Inhibition of these signal transduction pathways, as well as of tumor angiogenesis, adhesion mechanisms or apoptosis induction, can be the action point of these new molecules (summarized in Table 2, see also [42]). 

**Table 2:** Small molecules and targeted therapies used in distant metastatic melanoma.

Agent	Target protein	Literature
**Receptor tyrosinase kinase (RTK) inhibitors**		
Imatinib, Dasatinib, Sunitinib, Erlotinib	RTKs	[43,44]
SUI1274	c-Met/HGF	[45]
**RAS-RAF-MEK-ERK signal pathway inhibitors**		
AZD6244	MEK1, 2	[46]
PD0325901	MEK1, 2	[47]
PLX4032, PLX4720	Mutant B-Raf	[48,49]
Sorafenib	B-Raf, c-Kit, C-Raf Flt-3, PDGF, VEGF-2 , VEGF -3	[50,51,52]
Tanespimycin	Hsp90, as well as B-Raf, Akt, and others)	[53]
Tipifarnib	Farnesyltransferase	[52]
**PI3K-AKT signal pathway inhibitors**		
Deforolimus, Everolimus, Temsirolimus	MTOR	[54,55]
Perifosine, PX-866	Akt	[56]
PI 103	PI3K/mTOR	[57]
SB216763	GSK3beta	[58]
XL765	PI3K/mTOR	[59]
**Targeting anti-apoptotic proteins**		
ABT-737	Bcl-2 group	[60]
Obatoclax mesylate (GX15-070)	Bcl-2 group	[61]
Oblimersen sodium (G3139)	Bcl-2	[62]
YM155	Survivin	[63]
**Targeting the neovasculature**		
Bevacizumab	VEGF	[64,65]
Axitinib (AG-013736)	VEGF	[66]
**Others**		
Bortezomib (PS-341)	proteasome	[67,68]
Elesclomol	oxidative stress induction	[69]

Sorafenib is a multikinase inhibitor with selectivity for B-Raf, C-Raf, VEGFR-2 and -3, platelet-derived growth factor receptor (PDGFR) and c-Kit. Because of the high rate of activating B-Raf mutations in melanoma, sorafenib is an agent to treat metastatic melanoma. As a single agent, it has been shown to stabilize the disease in 19% of metastatic melanoma patients [70]. In contrast to sorafenib, PLX4032 is a selective inhibitor of the oncogenic V600E mutant BRAF kinase. V600E *BRAF* is the most common kinase mutation in melanoma (60%). Promising results in distant metastatic melanoma were recently shown for this substance [71,72]. 

Another interesting target is the receptor tyrosine kinase KIT. Activating Kit mutations have been found in mucosal and acral-lentiginous melanomas. Targeting Kit by receptor tyrosine-kinase inhibitors (imatinib, dasatinib, sunitinib) have shown some dramatic responses in patients with very high c-KIT expression and/or documented activating mutations [73,74,75]. This fosters the belief that focused studies in patients selected on the basis of c-KIT mutational status will yield more encouraging results.

## 5. Conclusion

The prognosis of patients with metastatic malignant melanoma remains poor. Only low response rates from 10% to 25% have been achieved by the most effective single-agent chemotherapies. More aggressive chemotherapy regimens (polychemotherapies) have reached response rates of about 40% but there was no significant benefit in overall survival compared to single-agent chemotherapies. Single-agent or combination chemotherapy, new agents or biologic response modifiers alone have not yet shown response rates of durable remissions that are high enough to affect median survival. There is an urgent need for more effective agents and developing new innovative treatment options to achieve higher response rates in treatment of metastatic melanoma. There are many opportunities for improving the treatment of patients with melanoma, including novel immunotherapy approaches, molecular targeted and antiangiogenic therapies. Further efforts are needed to select treatments for patients based on tumor and host molecular and genetic features. These new substances need to be evaluated in clinical trials. 

## Declaims

None of the authors has financial or proprietary interests in any material or method mentioned.
